# Increased expression of MARCH8, an E3 ubiquitin ligase, is associated with growth of esophageal tumor

**DOI:** 10.1186/s12935-017-0490-y

**Published:** 2017-12-04

**Authors:** Shivam Singh, Anoop Saraya, Prasenjit Das, Rinu Sharma

**Affiliations:** 1 University School of Biotechnology, Guru Gobind Singh Indraprastha University, Sector-16(C), Dwarka, Delhi, 110078 India; 2 Department of Gastroenterology, All India Institute of Medical Sciences, Ansari Nagar, New Delhi, 110029 India; 3 Department of Pathology, All India Institute of Medical Sciences, Ansari Nagar, New Delhi, 110029 India

**Keywords:** Esophageal squamous cell carcinoma, Immunohistochemistry, MARCH8, RNA interference, Migration/invasion, Apoptosis

## Abstract

**Background:**

Herein, for the first time, we report aberrant expression of membrane-associated RING-CH8 (MARCH8) in human esophageal squamous cell carcinoma. MARCH8 is a member of the recently discovered MARCH family of really interesting new genes (RING) E3 ligases. Though initial studies primarily focused on its immunomodulatory role, the newly discovered targets of this E3 ligase point towards its possible role in other biological processes such as embryogenesis and inhibition of apoptosis. However, its relevance in cancers is yet to be elucidated.

**Methods:**

We carried out quantitative real time PCR and immunohistochemistry to examine the levels of MARCH8 mRNA and protein in esophageal squamous cell carcinoma tissues. The role of MARCH8 in esophageal cancer cells was evaluated by cell proliferation, clonogenic and migration/invasion assays and flow cytometry with MARCH8 gene knockdown.

**Results:**

Significantly increased expression of MARCH8 mRNA was found in esophageal squamous cell carcinoma as compared to distant matched non-malignant tissues (*p* = 0.024, AUC = 0.654). Immunohistochemical analysis revealed overexpression of MARCH8 protein in 86% of esophageal squamous cell carcinoma tissues (*p* < 0.001, AUC = 0.908). Interestingly, intense nuclear staining of MARCH8 protein was detected in cancer cells in addition to its cytoplasmic expression. Knockdown of MARCH8 resulted in decreased proliferation, migration, invasion and clonogenic potential of esophageal cancer cells. In addition to this, silencing of MARCH8 induced apoptosis in esophageal cancer cells which was measured by cell cycle distribution assay which showed increase in sub G0 and G2/M populations (cell death) and decrease in S-phase population. To further check the type of apoptosis induced by MARCH8 silencing, annexin assay was performed which showed significant increase in the number of cells in early apoptotic phase.

**Conclusions:**

Overall, increased expression of MARCH8 gene in preneoplastic and neoplastic esophageal tissues and its knockdown effect on cancer cell properties demonstrated herein points towards the potential role of this protein in esophageal tumorigenesis.

## Background

Esophageal cancer is the eighth most common cancer worldwide and the sixth-leading cause of cancer-related deaths [1]. The molecular mechanism underlying tumor formation and progression is still not fully illuminated. Over the last decade, E3 ubiquitin ligases, enzymes that confer specificity to the ubiquitination process, have been shown to play a significant role in carcinogenesis [2–4].

Membrane-associated RING-CH (MARCH) protein family is comprised of 11 gene members among which MARCH8 was the first mammalian MARCH protein to be discovered. Overexpression of MARCH8 has been reported to downregulate immunomodulatory proteins viz. MHC II, B7-2, TfR (transferrin receptor), CD166, CD44, CD88, CD98, IL1RAP and syntaxin 4 [5–11]. MARCH8 mediated downregulation of TNF-related apoptosis inducing ligand receptor 1 (TRAIL-R1) has been shown to prevent breast cancer cells from undergoing apoptosis suggesting it to be a potential target for knockdown studies which may provide therapeutic benefit to patients suffering from cancer [12]. A recent study revealed the role of MARCH8 in embryogenesis wherein, overexpression of MARCH8 led to decreased surface expression of E-cadherin in zebrafish and *Xenopus* leading to loss of cell adhesion and abnormal cell migration [13, 14]. In addition to these reports, Kumar et al. identified MARCH8 as one of the differentially expressed gene in esophageal squamous cell carcinoma (ESCC) using 19.1K cDNA microarrays [15]. However, its expression and clinical relevance in ESCCs has not yet been analysed.

In the present study, we have reported aberrant expression of MARCH8 gene in esophageal squamous cell carcinoma (ESCC). Moreover, we have analysed the role of MARCH8 gene in ESCC. We observed that silencing of MARCH8 affects proliferation, migration/invasion, colony formation potential and apoptosis of ESCC cells.

## Methods

### Study subjects

Thirty-five cancerous and distant matched non-malignant tissue (5 cm apart from tumor) biopsies were collected from patients with ESCC who underwent endoscopy at Department of Gastroenterology, AIIMS. One part of the tissue taken in 10% formalin and embedded in paraffin was used for hematoxylin/eosin staining and immunohistochemical analysis. The clinicopathological data were recorded in a predesigned performa that included site of lesion, histopathological differentiation, age, gender, nature of diet, tea, alcohol and tobacco consumption, and family history. The sites of esophageal squamous cell tumors included upper, mid and lower esophagus.

### Cell culture and transfections

Human esophageal carcinoma cell line, KYSE-410 (ECACC 94072023), was obtained from Sigma-Aldrich (Bangalore, India). The cells were grown in RPMI-1640 media supplemented with 10% heat inactivated fetal bovine serum (FBS) and 1% antibiotics in a 5% carbon dioxide and 37 °C atmosphere. KYSE-410 cells were transfected with 50 nmol/l MARCH8 siRNA (5′-AAUGACUCAUGAAAUGUCC-3′, Ambion, CA, USA) or scrambled sequence siRNA (Ambion) using Lipofectamine 3000 (Invitrogen, CA, USA) as transfecting agent in a serum- and antibiotics-free medium.

### Quantitative real time PCR (qRT-PCR)

Total RNA was extracted from cell line, ESCC and distant matched non-malignant tissues using RNAeasy mini kit (Qiagen, Copenhagen, Denmark) as per the manufacturer’s protocol. cDNA was synthesized from 1 µg of total RNA by reverse transcription PCR. To prevent genomic DNA amplification, primers were designed from exon–exon junction. The details of primer sequences are given in Table 1. A two-step real time PCR, for analysing the expression of MARCH8 mRNA, was performed as described before [16].

**Table 1:** Primer sequences for qRT-PCR

S. no.	Gene	Sequences (5′ to 3′)	Annealing temperature (°C)	Product size (bp)
1.	MARCH8	TGCATCAGATCTCTGCCATT TGGACGTCATCTGCAACTTC	56	435
2.	5s rRNA	GTCTACGGCCATACCACCCTG AAAGCCTACAGCACCCGGTAT	60	121

### Immunohistochemistry

Paraffin-embedded sections (5 µm) of histologically confirmed human esophageal normal (n = 25) and ESCC (n = 35) tissues were obtained on poly-l-lysine coated slides. Briefly, the tissue sections were deparaffinized and rehydrated. Tris–EDTA buffer (10 mM Tris-base, 1 mM EDTA, pH 9.0) was used for carrying out antigen retrieval. To quench endogenous peroxidase activity, sections were incubated with 0.3% v/v hydrogen peroxide in methanol for 30 min, followed by blocking in 1% normal horse serum to prevent non-specific binding. After this, slides were incubated overnight with 1:50 diluted rabbit polyclonal anti-MARCH8 antibody of human origin (Santa Cruz Biotechnology, Inc., Dallas, TX, USA) at 4 °C. Next day, the slides were washed and coated with HRP conjugated anti-rabbit IgG [ImmPRESS anti-rabbit Ig (peroxidase) Polymer Detection kit, Vector Laboratories Inc, USA] for 30 min at RT. The colour was developed using diaminobenzidine as the chromogen. Haematoxylin was used for nuclear staining. ESCC tissue sections not treated with anti-MARCH8 antibody were used as negative controls. For MARCH8 protein expression, sections were counted as positive if epithelial cells showed immunopositivity in the nucleus/cytoplasm when observed independently by three of us (SS, RS and PD). The slides were scored based on the percentage of immunostained cells as ≤ 10% = 0; 11–20% = 1; 21–40% = 2; 41–60% = 3; 61–80% = 4 and > 81% = 5. Slides were also scored on the basis of staining intensity as faint = 1; moderate = 2 and strong = 3. Finally a total score was found by adding the scores of percentage positivity and intensity. If any disagreement in the grading between the 3 investigators occurred, those slides were reviewed by all three of us and unanimity was reached by discussion. Based on sensitivity and specificity, calculated by ROC curve analysis, a total score cut-off value of 4 was defined as MARCH8 immunopositivity.

### In-silico prediction of MARCH8 protein subcellular localization

Subcellular localization of MARCH8 protein was predicted using CELLO v.2.5 database (http://cello.life.nctu.edu.tw/) which is a multi-class SVM classification system. CELLO database uses four types of schemes: amino acid composition, dipeptide composition, partitioned amino acid composition and sequence composition based on physicochemical properties of amino acids [17]. In addition to this, cNLS Mapper database (http://nls-mapper.iab.keio.ac.jp/cgi-bin/NLS_Mapper_form.cgi) was also used to predict nuclear localization signals (NLS) in MARCH8 protein sequence [18].

### Protein isolation and western blotting

For total protein isolation, the KYSE-410 cells were lysed in RIPA buffer (Sigma) and scraped off the dish. For nuclear and cytoplasmic protein extraction, cells were lysed using Cytoplasmic and Nuclear Protein Enrichment kit (Amresco, Ohio, USA) according to manufacturer’s protocol. The protein quantification was carried out by Bradford method (BioRad, USA) using BSA as a standard. Total cellular proteins as well as nuclear and cytoplasmic protein fractions (100 µg protein/lane) were resolved on 12% sodium dodecyl sulphate-poly-acrylamide gels, transferred to PVDF membranes and immunolabelled with rabbit polyclonal anti-GAPDH (Glyceraldehyde-3-Phosphate Dehydrogenase, 1:200 dilution, Santa Cruz Biotechnology), mouse monoclonal anti-proliferating cell nuclear antigen or PCNA (DAKO A/S Copenhagen, Denmark, 1:500 dilution) and anti-MARCH8 (1:100 dilution) antibodies in 1% bovine serum albumin for 1 h at room temperature. PVDF membranes were incubated with respective horseradish peroxidase (HRP)-tagged secondary antibodies. The HRP-tagged immunolabelled proteins were detected by Enhanced Chemiluminescence kit (Thermo Scientific, USA). The integrated density values (IDV) for each group were determined using Image J software (https://imagej.nih.gov) and normalization was done by dividing the corrected IDV of MARCH8 protein in each group by corrected IDV of GAPDH protein in the corresponding group.

### Confocal and immunofluorescence microscopy

To determine the localization of MARCH8 protein in cancer cells (KYSE-410), confocal and immunofluorescence microscopy were performed. Briefly, the cells were fixed in methanol at – 20 °C for 20 min and permeabilized with 0.5% Triton X-100 in 1X-PBS for 10 min. To prevent non-specific binding, the cells were blocked with 3% BSA and 0.05% Triton X-100 in PBS for 1 h. Then the cells were incubated with rabbit polyclonal anti-MARCH8 antibody of human origin (1:50 dilution) overnight at 4 °C, followed by incubation with Alexa Fluor 488-conjugated goat anti-rabbit secondary antibody (Invitrogen) at room temperature for 30 min (1:1000 dilution). The cells were observed under a confocal microscope (Leica TCS SP2 AOBS), after counterstaining of the nuclei with DAPI for confocal microscopy and under inverted fluorescence microscope (Nikon), after nuclei counterstaining with propidium iodide (PI) for immunofluorescence microscopy.

### MTT assay

Cell growth was analyzed by 3-(4,5-dimethylthiazolyl-2)-2,5-diphenyltetrazolium bromide (MTT) assay. 1 × 10^4^ KYSE-410 cells were seeded in a 96-well plate and transfected with siRNA after 24 h. Cell growth inhibition rate was determined at 24, 48 and 72 h. The cell growth inhibition rate was calculated with the equation: inhibition rate  =  (1 − ODtreatment/ODcontrol) × 100. The results were expressed as the percentage of inhibition relative to control cells.

### Colony formation assay

1000 KYSE-410 cells transfected with MARCH8 siRNA/scrambled sequence siRNA and untransfected cells were cultured in 6-well plates for 1 week to check the number of colonies formed using 0.5% (w/v) crystal violet in distilled water.

### Scratch assay

Scratch assay was performed to test the effect of MARCH8 silencing on cell migration. For Scratch assay, 4 × 10^5^ cells were seeded on a 6-well plate overnight. After 24 h post-transfection, a scratch was made using a pipette tip. At 0, 24 and 48 h after scratching, cells were observed and images were taken at 4× magnification in inverted fluorescence microscope (Nikon). The migration area among different groups was measured as the average percent of wound closure as compared to that at 0 h.

### Boyden chamber assay for cell migration and invasion

Boyden chamber assay was also performed to check the effect of MARCH8 knockdown on KYSE-410 cell migration and invasion. Briefly, 48 h post-transfection, 4 × 10^4^ cells, re-suspended in 1× PBS, were seeded on the upper side of the Boyden chamber (SPL Lifesciences Co. Ltd., Korea) containing polycarbonate membrane filter (6.5 mm diameter; 8 µm pore size). Unlike migration, Matrigel basement membrane matrix (Corning, Massachusetts, USA) was used to coat the upper side of Boyden chamber to assess the effect of MARCH8 knockdown on invasive capacity of KYSE-410. Similar to migration assay, cells were added over the Matrigel on the upper side of Boyden chamber. The lower chamber was filled with a complete medium. Cells were incubated for 24 h, and those cells that did not migrate through the pores were detached using a cotton swab. Cells on the lower surface of the membrane were fixed in prechilled methanol, stained with 1 µg/µl of DAPI and counted by an inverted fluorescence microscope (magnification, 100×, Nikon). The mean number of cells migrated/invaded was calculated by dividing the average number of cells in each of the random fields within an insert by the area of the microscopic viewing field and then multiplied by the entire area of the Boyden chamber.

### Cell cycle assay

1 × 10^5^ cells, seeded in 6-well plates, were transfected with either MARCH8 siRNA or scrambled sequence siRNA. After 72 h, the cells were harvested and fixed in 70% ethanol overnight at − 20 °C. Next day, the cell pellet was resuspended in 10 µg/ml of PI and cell cycle distribution was analysed with LSR II flow cytometer (Becton–Dickinson) and the FACSDiva software-version 6.1.3 (Becton–Dickinson).

### Apoptosis assay

To detect apoptosis, phycoerythrin (PE) Annexin V Apoptosis Detection kit I (BD Pharmingen, California, USA) was used. Fluorescence was detected using LSR II flow cytometer. Cells treated with 6 µg of camptothecin for 10 h and untreated cells, respectively, served as positive and negative controls for dual staining.

### Statistical analysis

Data was statistically analyzed by using Statistical Program for Social Sciences (SPSS) software, version 17.0 (SPSS Inc., Chicago, IL, USA). Each experiment was performed in triplicates and the results were represented as mean ± SD using either Student’s t test or ANOVA. Mann–Whitney U test was applied to evaluate statistical differences in MARCH8 mRNA expression between ESCC and distant matched non-malignant tissue populations. The associations between clinicopathological parameters of ESCC patients and MARCH8 protein expression were examined by χ^2^ test. A *p* ≤ 0.05 was considered as a criterion for statistical significance.

## Results

### MARCH8 gene expression in ESCC

**Fig. 1:**
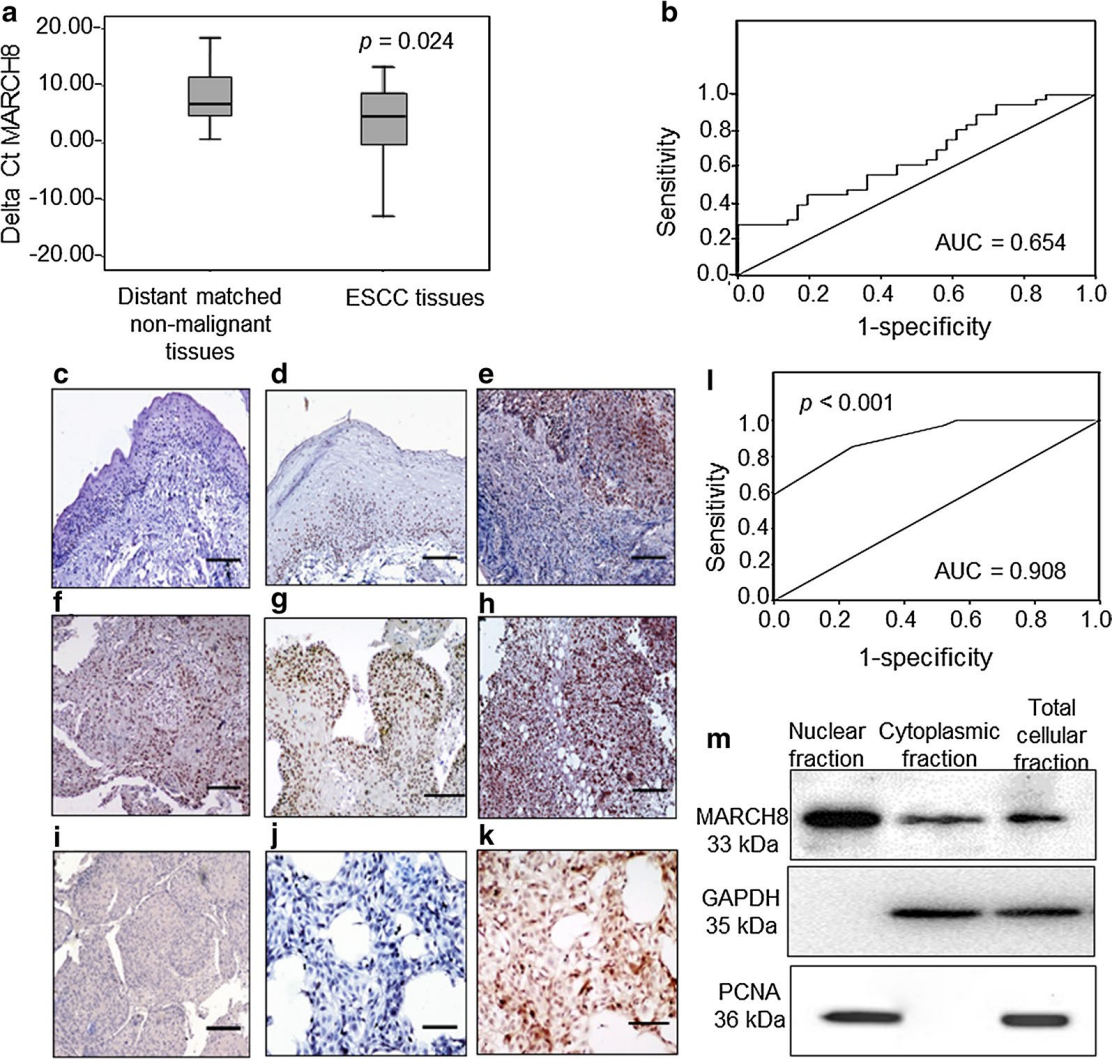
Increased expression of MARCH8 in ESCC tissues. **a** Boxplot diagram displaying differential expression of MARCH8 mRNA in cancer versus distant matched non-malignant tissues. **b** Receiver operating characteristic curve of MARCH8 for discriminating ESCC tissues from distant matched non-malignant tissues. **c**–**k** Photomicrograph showing the expression of MARCH8 protein in esophageal tissue sections. **c** Shows no detectable MARCH8 staining in distant matched non-malignant tissue. **d** Shows nuclear staining in hyperplastic esophageal tissue. **e** Dysplastic lesion showing nuclear MARCH8 protein. **f** Well-differentiated SCC showing nuclear MARCH8 staining. **g** Moderately-differentiated SCC showing predominantly nuclear and faint cytoplasmic MARCH8 staining. **h** Poorly-differentiated SCC showing both nuclear and cytoplasmic staining. **i** Represents the negative control in which the ESCC tissue section was not treated with primary rabbit anti-MARCH8 antibody. **j** Shows negative control KYSE-410 cells wherein no primary antibody was added. **k** KYSE-410 cells showing MARCH8 protein expression in the cytoplasm and nucleus of the cells. **l** ROC analysis for MARCH8 protein expression in ESCC tissues. **m** Western blot for MARCH8 protein detection in nucleus as well as cytoplasmic fractions. Size bar = 100 µm (**c**–**k**)

MARCH8 expression was determined in ESCC tissues and distant matched non-malignant esophageal tissues using qRT-PCR. Significantly upregulated expression of MARCH8 was found in 23 (66%) out of 35 ESCC patients (*p* = 0.024). Lower delta C_t_ values are indicative of higher MARCH8 expression (Fig. 1a, b). No significant correlation was observed between MARCH8 mRNA expression in tissues and clinicopathological parameters.

### MARCH8 protein expression in ESCCs, dysplasia and distant matched non-malignant tissues

The immunohistochemistry results revealed high percentage positivity and increased intensity of MARCH8 staining in 30/35 (86%) ESCC tissues as compared to distant matched non-malignant tissues which either did not show any detectable staining for MARCH8 protein or a very faint staining in lesser proportion of cells in the tissues was observed (Fig. 1c–h, *p* < 0.001, Table 2). MARCH8 expression was observed in nucleus as well as cytoplasm of esophageal cancer tissues. Interestingly, all the preneoplastic tissues showed overexpression of MARCH8 protein suggesting its possible role in early stages of esophageal carcinogenesis (Fig. 1d, e). No immunostaining was observed in the negative control, in which the ESCC tissue section and KYSE-410 cells were not treated with primary rabbit anti-MARCH8 antibody (Fig. 1i, j). In addition to this, immunocytochemical analysis revealed MARCH8 protein expression in both nucleus and cytoplasm of KYSE-410 cells (Fig. 1k). Receiver operating characteristic (ROC) curve analysis revealed an area under the curve (AUC) of 0.908 with sensitivity of 85% and specificity of 76% (Fig. 1l). No correlation between MARCH8 expression and clinicopathological variables was observed (Table 2).

**Table 2:** Relationship of MARCH8 protein expression with clinicopathological parameters of ESCC patients

Clinicopathological parameters	Total cases	MARCH8 protein positivity n (%)	*p* value
Distant matched non-malignant	25	6 (24)	
ESCC	35	30 (86)	< 0.001
Age (years)			
< 40	10	7 (70)	1.000
≥ 40	25	23 (92)	
Gender			
Male	20	19 (95)	0.624
Female	15	11 (73)	
Histopathology grading			
WDSCC	7	5 (71)	0.487
MDSCC	18	16 (88)	
PDSCC	10	9 (90)	

### Subcellular localization prediction

As MARCH8 protein was found to be localized in the nucleus, in addition to cytoplasm, of ESCC tissues during immunohistochemical analysis, we were interested in looking for presence of any nuclear localization signals (NLS) in its protein sequence. Firstly, to predict the subcellular localization of MARCH8, CELLO database was used. CELLO predicts which of the 12 subcellular localizations in eukaryotes that the targeted protein might be found in, with the 12 eukaryotic localizations being chloroplasts, the cytoplasm, the cytoskeleton, the endoplasmic reticulum, the extracellular/secretory space, the Golgi, lysosomes, mitochondria, the nucleus, peroxisomes, the plasma membrane, and vacuoles. Among these, MARCH8 was predicted to have plasma membrane, extracellular and nuclear localization (Table 3). In order to check the presence of NLS in MARCH8 protein sequence, cNLS Mapper database was used (cut-off score = 3.0). It predicted the presence of three bipartite NLSs in MARCH8 protein sequence (Table 4).

**Table 3:** CELLO results

CELLO prediction	Localization	Reliability
1.	Plasma membrane	1.874*
2.	Extracellular	1.248*
3.	Nuclear	1.234*
4.	Cytoplasmic	0.338
5.	Mitochondrial	0.152
6.	Chloroplast	0.069
7.	Golgi	0.022
8.	Peroxisomal	0.020
9.	Vacuole	0.014
10.	Lysosomal	0.011
11.	ER	0.011
12.	Cytoskeletal	0.007

* The most reliable sub-cellular localizations of the MARCH8 protein

**Table 4:** Predicted bipartite NLS

Position	Sequence	Score
23	RSKTKEKEREEQNEKTLGHFMSHSSNISKAGSPP	3.8
114	WIKSSDTRCCELCKYEFIMETKLKPLRKWE	3.0
225	LWKRLKAYNRVIYVQNCPETSKKNIFEK	3.7

### MARCH8 protein localization in esophageal cancer cells

Western blot analysis was performed to confirm the presence of MARCH8 protein in nuclear and cytosolic compartments of the ESCC cells (KYSE-410). Figure 1m shows the MARCH8 expression (33 kDa) in nuclear, cytoplasmic and total protein fractions of KYSE-410 cells. These results validate that, in addition to the cytoplasmic localization, human MARCH8 protein localizes in the nucleus of esophageal cancer cells (KYSE-410).

**Fig. 2:**
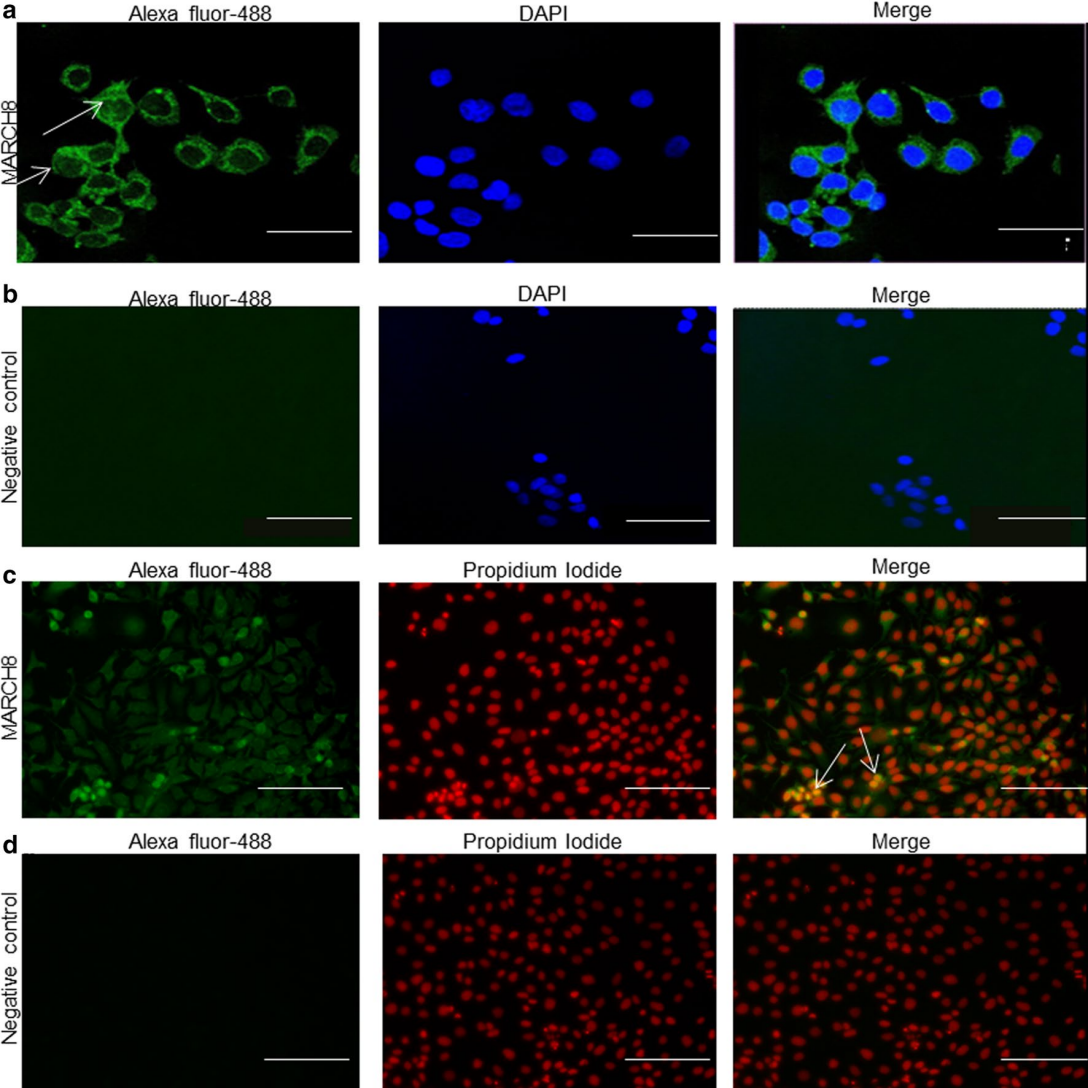
Nuclear and cytoplasmic localization of MARCH8 protein in KYSE-410 cells. Cells were incubated with anti-MARCH8 antibody which was visualized with Alexa Fluor-488 conjugated secondary antibody and counterstained with either DAPI (**a**, **b**) or PI (**c**, **d**) for nuclear staining. Nuclear localization was recognized in the merge image by either cyan staining produced by the blending of green signal from Alexa Fluor-488 and blue signal from DAPI (**a**) or yellow staining by merging of green signal from Alexa Fluor-488 and red signal from PI (**c**). **b**, **d** Represent negative control wherein no primary antibody was added. Size bar = 45 µm (**a**, **b**) and 100 µm (**c**, **d**)

In order to further confirm the localization of MARCH8 protein in ESCC cells (KYSE-410), confocal and immunofluorescence microscopy were carried out. MARCH8 was found to be expressed both in the nucleus and cytoplasm of esophageal cancer cells (Fig. 2a, c). It is noteworthy that intense staining of MARCH8 was clearly observed in nuclear and perinuclear region of the cells.

### MARCH8 gene silencing

**Fig. 3:**
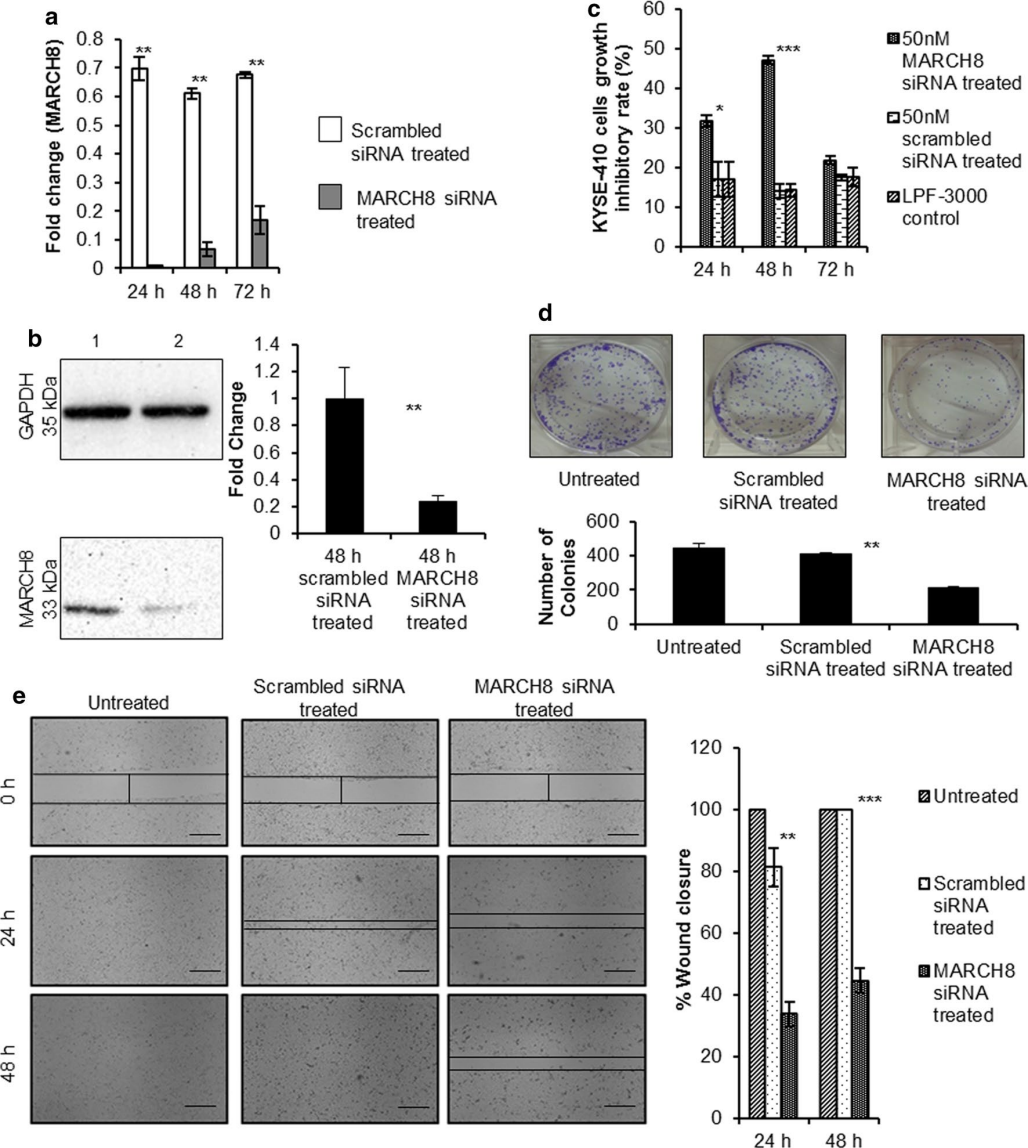
**a** Relative expression of MARCH8 mRNA in MARCH8 (50 nmol/l) silencing group and negative control group (*p < 0.05). **b** Western blot showing downregulation of MARCH8 protein after 48 h of transfection with 50 nmol/l of MARCH8 siRNA (1: scrambled siRNA treated and 2: MARCH8 siRNA treated); **p = 0.01. **c** Cell growth Inhibitory assay. The inhibitory rate of MARCH8 siRNA treated cells was higher than scrambled siRNA treated cells (*p ≤ 0.05 and ***p < 0.001). **d** Colony formation assay. The number of colonies formed by MARCH8 siRNA treated cells were 215 ± 6.36 (mean ± SD) as compared to 412 ± 6.36 (mean ± SD) in scrambled siRNA treated or 444 ± 26.16 (mean ± SD) in untreated cells (**p < 0.01). **e** Scratch assay to detect migration ability of KYSE410 cells. The wound healing capacity of MARCH8 siRNA treated cells was lower than scrambled siRNA treated and untreated cells (**p < 0.01 and ***p < 0.001). Size bar = 500 µm

Assessment of MARCH8 mRNA levels at 24, 48 and 72 h post-transfection showed a significant reduction of 69% (*p* = 0.0018), 59% (*p* = 0.0014) and 51% (*p* = 0.0045), respectively, in MARCH8 silencing group as compared to negative control group (Fig. 3a). In addition to this, 74% reduction was observed in its protein levels after 48 h of siRNA transfection in MARCH8 silencing group as compared to negative control group as shown in Fig. 3b (*p* = 0.005).

### Silencing of MARCH8 increases the cell growth inhibitory rate

To detect the effect of MARCH8 gene knockdown on cell growth inhibition, MTT assay was carried out in MARCH8 silencing group, negative control group, transfection reagent only control group and blank control group (Fig. 3c). The data was measured from day 1 to day 3 post-transfection. The inhibitory rate of MARCH8 silencing group was found to be highest (33%) at 48 h post-transfection as compared to negative control group (*p* = 0.000). Moreover, a significantly reduced clonogenic potential of KYSE-410 cells after MARCH8 silencing was observed (Fig. 3d, *p* = 0.002).

### MARCH8 siRNA suppresses wound healing in esophageal cancer cells

Migration of cancer cells for tissue invasion is required for progression of tumours. To check whether MARCH8 silencing modifies migration of KYSE-410 cells, scratch assay was performed. Wound closure was evaluated at different time points viz. 24 and 48 h post scratching. 48 h were required for the wound to completely close up in untreated cells. At 24 and 48 h post MARCH8 siRNA transfection, a significant difference was found between the groups (*p* = 0.001 and *p* = 0.000, respectively). Tukey’s multiple comparisons test revealed that cell migration was significantly inhibited at 24 and 48 h after transfection of MARCH8 siRNA as compared to the negative control group (*p* = 0.003 and *p* = 0.00, respectively). MARCH8 siRNA treatment inhibited cell migration in a time dependent manner reducing the wound closure to only 33.99 ± 3.92% at 24 h and 44.77 ± 4.11% at 48 h as compared to the negative control where scratch healed up to 81.41 ± 6.25% (at 24 h) and 100% (at 48 h) (Fig. 3e).

### MARCH8 silencing resulted in decreased migration and invasion potential of esophageal cancer cells

**Fig. 4:**
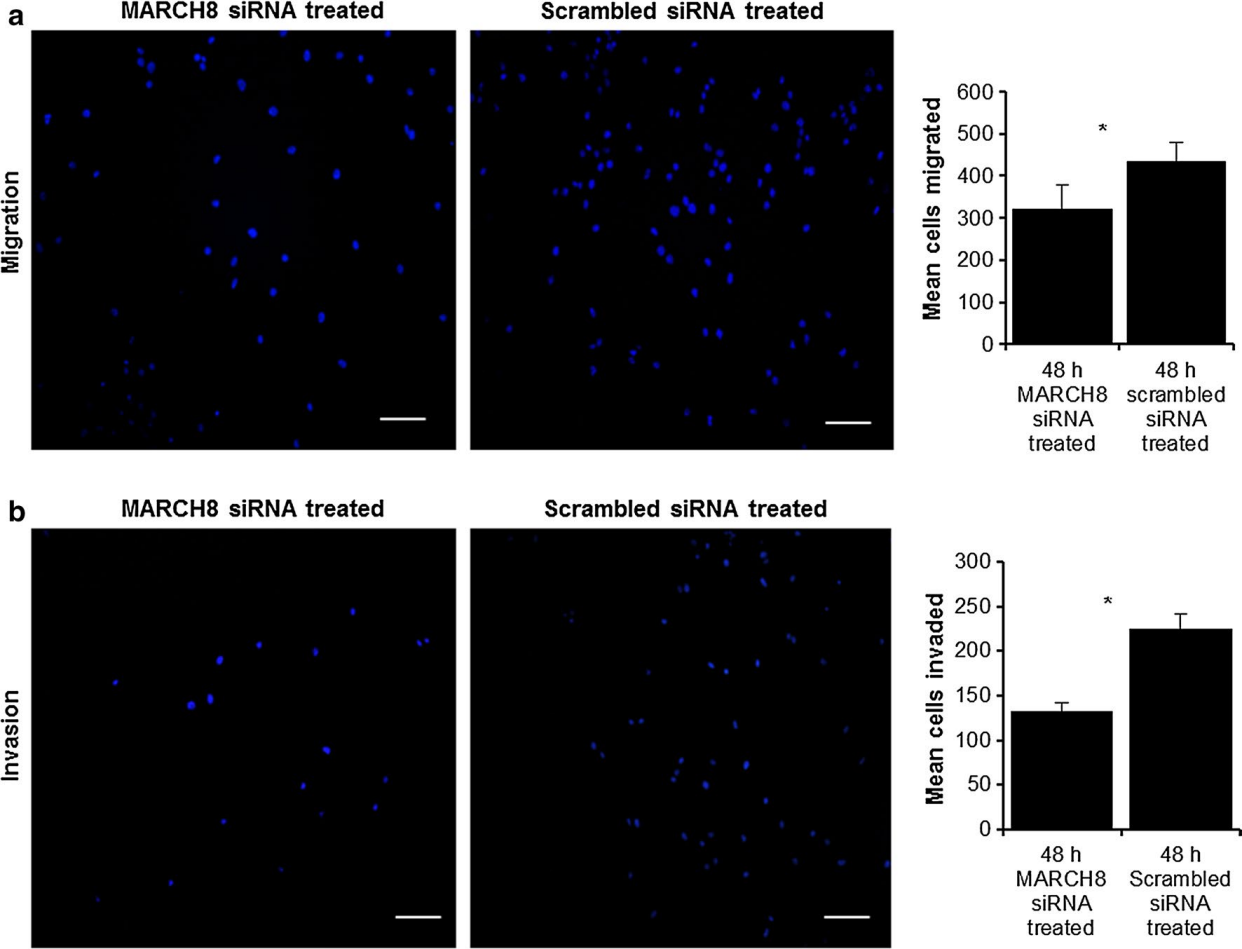
Boyden chamber cell migration assay. **a** The migration of MARCH8 siRNA-treated cells was lower as compared to scrambled siRNA-treated cells (*p < 0.05). Matrigel invasion assay (**b**). Significantly lower invasion capacity was observed in MARCH8 siRNA treated cells as compared to scrambled siRNA treated cells (*p < 0.05). Size bar = 100 µm

We further evaluated the effect of MARCH8 siRNA on migration of KYSE-410 cells using Transwell assay. MARCH8 siRNA resulted in significantly decreased migratory ability of KYSE-410 cells at 48 h post-transfection (*p* = 0.05). The numbers of KYSE-410 cells that had migrated through the chamber were 321 ± 58 and 434 ± 45 in MARCH8 siRNA treated and negative control groups respectively (Fig. 4a).

As breakdown of extracellular matrix is a critical determinant of metastasis, invasion is more closely linked to the metastasis process. Hence, a modified Boyden chamber coated with matrigel was used to check the MARCH8 silencing effect on invasiveness of KYSE-410 cells. A significantly reduced invasion by KYSE-410 cells was observed in MARCH8 silencing group as compared to negative control group (Fig. 4b; *p* = 0.034).

### Downregulation of MARCH8 by siRNA affects cell cycle distribution and induces apoptosis

**Fig. 5:**
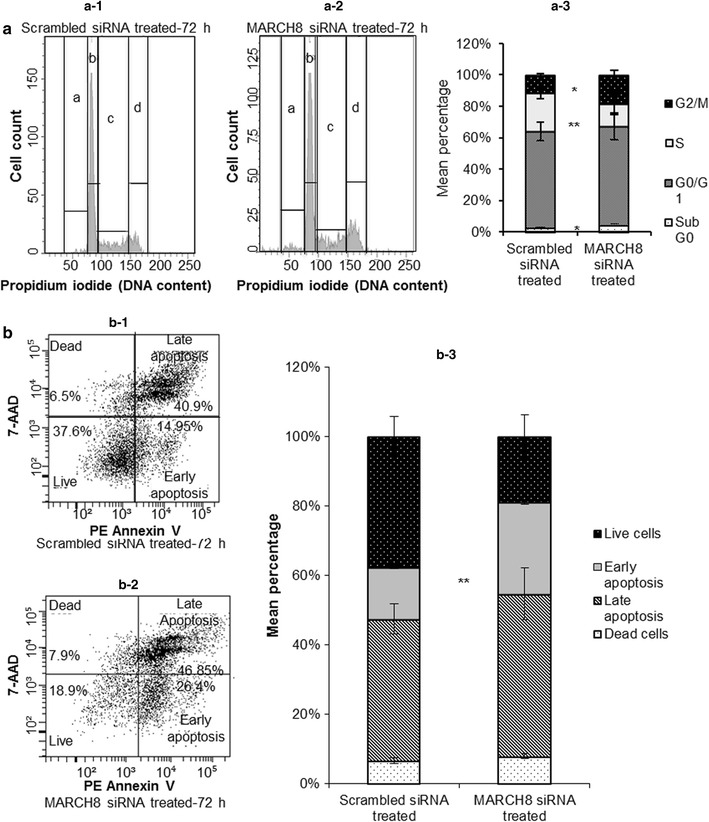
**a** Cell cycle analyses. **a-1** Shows DNA histograms of scrambled siRNA treated cells for 72 h and **a-2** shows DNA histograms of MARCH8 siRNA treated cells indicating increased cell death, lesser percentage of S-phase cells and more percentage of cells in G2/M phase in MARCH8 knockdown cells. **a-3** Stacked bar graph illustrates cell cycle distribution (**p < 0.01 and *p < 0.05). **b** Knockdown of MARCH8 induces late apoptotic changes and death in KYSE-410 cells (**b-2**) as compared to scrambled siRNA treated cells (**b-1**). **b-3** Shows percentage of apoptotic cells in scrambled siRNA treated cells and MARCH8 siRNA treated cells as mean ± SD (*p < 0.05). a: Sub G0 phase; b: G0/G1 phase; c: S-phase and d: G2/M phase

Increased cancer cell growth inhibitory rate assessed by MTT assay in MARCH8 silencing group can be result of increased apoptosis. Hence, cell cycle distribution was measured (Fig. 5a). Knockdown of MARCH8 resulted in an increase (3.69%, *p* = 0.015) in sub G0 population, decrease (11.57%, *p* = 0.002) in S-phase population and increase (14.43%, *p* = 0.028) in G2/M population as compared to negative control group (2.2, 20.2 and 9.51%, respectively).

In order to evaluate the type of cell death induced by silencing of MARCH8, Annexin assay was carried out (Fig. 5b). 72 h post-transfection a significant increase in the number of cells was observed in early apoptotic phase in MARCH8 silencing group (26.4%, PE Annexin-V positive and 7-AAD negative; *p* = 0.002) as compared to negative control group (14.95%).

## Discussion

Initial studies of MARCH8 primarily focused on its immunomodulatory role. But, its relevance in cancers has not yet been fully elucidated. In this study, we showed overexpression of MARCH8 in esophageal cancer tissues and its silencing inhibited proliferation, migration, invasion and clonogenicity of EC cells. Additionally, MARCH8 silencing enhanced the apoptosis of EC cells.

Immunohistochemical analysis revealed an increased expression of MARCH8 in ESCC tissues as compared to distant matched non-malignant tissues. Moreover, all the dysplastic tissues showed increased MARCH8 protein expression suggesting its possible involvement in early stages of esophageal cancer. Interestingly, MARCH8 expression was found to be localized in nucleus of ESCC cells in addition to its cytoplasmic expression. Earlier, MARCH8 expression has been shown to be localized in early endosomes and late endosomes [5, 8]. However, most of the MARCH8 protein subcellular localization information available till date relies on its ectopic expression in cells which may be one of the reasons that expression of MARCH8 in the nucleus was not reported earlier. In the present study, we have used antibodies to detect endogenous MARCH8 expression which offers more direct evidence of endogenous MARCH8 localization in the cell. Confocal microscopy and western blotting in ESCC cells further validated our immunohistochemical results. In addition to this, well-recognized nuclear localization signals were also found in MARCH8 protein sequence. However, further in-depth studies are necessary to examine the translocation of MARCH8 to the nucleus and its functional implication there.

Our results also demonstrated the effect of MARCH8 knockdown on cellular migration, invasion, growth inhibitory rate, and clonogenic potential of KYSE-410 cells. We found that inhibiting the MARCH8 expression resulted in higher growth inhibition of cells. To find out if this growth inhibition was related to cell cycle, DNA cell cycle analysis was carried out. It was observed that MARCH8 gene knockdown induces a significant increase in sub G0 and G2/M populations (cell death) and decreases the S-phase population suggestive of induced apoptosis due to MARCH8 silencing. To check this, apoptosis assay was performed which showed significantly more number of cells in early apoptotic phase after MARCH8 gene knockdown. This may be due to the fact that MARCH8 targets proteins that play significant role in apoptosis (Fas and TRAIL-R1) [8, 12]. TRAIL receptor signaling has been associated with metastasis suppression [19]. Van et al. [12] demonstrated that a unique membrane-proximal lysine in the cytoplasmic tail of TRAIL-R1 interacts with MARCH8 which ubiquitinates TRAIL-R1 and thus diminishes its steady-state cell surface expression which suggests MARCH8 as a potential determinant for tumor cell sensitivity to TRAIL receptor-targeted therapy [12].

Knockdown of MARCH8 was also found to suppress cancer cell properties like invasion, migration and colony formation ability of KYSE-410 cells. E-cadherin is a tumor-suppressor gene that prevents migration/invasion of the epithelial tumor cells [20]. Moreover, change in the surface levels of E-cadherin due to E3 ubiquitin ligase activity of MARCH8 has been reported to reduce adherence of the cells in the animal cap during embryogenesis in zebrafish [13]. In addition to this, MARCH7 which has recently been reported to be oncogenic was also found to regulate E-cadherin protein levels in ovarian cancer cells [21]. This suggests that downregulation of E-cadherin by MARCH8 may also contribute to cancer development as loss of E-cadherin-mediated adhesion system has been well documented to be involved in malignant transformation of the cells [22]. Moreover, most of the targets of MARCH8 have been found to be involved in immune modulation such as MHC II, B7.2, IL1RAP and TNF-α [6, 8, 11, 23]. However, role of MARCH8 in immune surveillance is yet to be analysed. Besides this, clinical and functional implications of other MARCH proteins viz. MARCH7, 1 and 5 have also been reported in ovarian cancer [19, 24, 25]. This suggests that MARCH gene silencing inhibits the aggressive behaviour of ESCC, indicating that this protein family may act as a potential therapeutic target for ESCC.

## Conclusions

In conclusion, our study provides first clinical and functional evidence of MARCH8 in ESCCs. Increased expression of MARCH8 gene in preneoplastic and neoplastic esophageal tissues and its knockdown effect on cancer cell properties demonstrated herein points towards the potential role of this protein in esophageal tumorigenesis.

## Abbreviations

MARCH8: membrane associated RING-CH 8
RING: really interesting new genes
ESCC: esophageal squamous cell carcinoma
qRT-PCR: quantitative real time PCR
TfR: transferrin receptor
TRAIL-R1: TNF-related apoptosis inducing ligand receptor 1
FBS: fetal bovine serum
MTT: 3-(4,5-dimethylthiazolyl-2)-2,5-diphenyltetrazolium bromide
SPSS: Statistical Program for Social Sciences
ROC: receiver operating characteristic
AUC: area under the curve
